# Antimicrobial susceptibility patterns and characterization of clinical isolates of *Staphylococcus aureus *in KwaZulu-Natal province, South Africa

**DOI:** 10.1186/1471-2334-6-125

**Published:** 2006-07-28

**Authors:** Adebayo O Shittu, Johnson Lin

**Affiliations:** 1 School of Biochemistry, Genetics, Microbiology and Plant Pathology, University of KwaZulu-Natal (Westville Campus), Private Bag X54001, Durban, Republic of South Africa; 2 Department of Microbiology, Obafemi Awolowo University, Ile-Ife, Nigeria

## Abstract

**Background:**

Antimicrobial resistance of *Staphylococcus aureus *especially methicillin-resistant *S. aureus *(MRSA) continues to be a problem for clinicians worldwide. However, few data on the antibiotic susceptibility patterns of *S. aureus *isolates in South Africa have been reported and the prevalence of MRSA in the KwaZulu-Natal (KZN) province is unknown. In addition, information on the characterization of *S. aureus *in this province is unavailable. This study investigated the susceptibility pattern of 227 *S. aureus *isolates from the KZN province, South Africa. In addition, characterization of methicillin-sensitive *S. aureus *(MSSA) and MRSA are reported in this survey.

**Methods:**

The in-vitro activities of 20 antibiotics against 227 consecutive non-duplicate *S. aureus *isolates from clinical samples in KZN province, South Africa were determined by the disk-diffusion technique. Isolates resistant to oxacillin and mupirocin were confirmed by PCR detection of the *mecA *and *mup *genes respectively. PCR-RFLP of the coagulase gene was employed in the characterization of MSSA and MRSA.

**Results:**

All the isolates were susceptible to vancomycin, teicoplanin and fusidic acid, and 26.9% of isolates studied were confirmed as MRSA. More than 80% of MRSA were resistant to at least four classes of antibiotics and isolates grouped in antibiotype 8 appears to be widespread in the province. The MSSA were also susceptible to streptomycin, neomycin and minocycline, while less than 1% was resistant to chloramphenicol, ciprofloxacin, rifampicin and mupirocin. The inducible MLS_B _phenotype was detected in 10.8% of MSSA and 82% of MRSA respectively, and one MSSA and one MRSA exhibited high-level resistance to mupirocin. There was good correlation between antibiotyping and PCR-RFLP of the coagulase gene in the characterization of MRSA in antibiotypes 1, 5 and 12.

**Conclusion:**

In view of the high resistance rates of MRSA to gentamicin, erythromycin, clindamycin, rifampicin and trimethoprim, treatment of MRSA infections in this province with these antibacterial agents would be unreliable. There is an emerging trend of mupirocin resistance among *S. aureus *isolates in the province. PCR-RFLP of the coagulase gene was able to distinguish MSSA from MRSA and offers an attractive option to be considered in the rapid epidemiological analysis of *S. aureus *in South Africa. Continuous surveillance on resistance patterns and characterization of *S. aureus *in understanding new and emerging trends in South Africa is of utmost importance.

## Background

*Staphylococcus aureus *has emerged as one of the most important human pathogens, and has over the past several decades, been a leading cause of hospital and community-acquired infections [1]. It is associated with a variety of clinical infections including septicemia, pneumonia, wound sepsis, septic arthritis, osteomyelitis and post-surgical toxic shock syndrome with substantial rates of morbidity and mortality [2-5]. One of the reasons for the success of this human pathogen is its great variability, occurring at different periods and places with diverse clonal types and antibiotic resistance patterns within regions and countries. Although infections caused by antibiotic-resistant *S. aureus *bring about serious problems in the general population, such infections can be particularly devastating for the very young, the elderly and the immunocompromised [6].

Antimicrobial resistance among nosocomial pathogens is a significant problem in many countries with severe consequences including increased medical costs, morbidity and mortality of patients [7]. Since the emergence of *S. aureus *strains with resistance to penicillin and methicillin in 1948 and 1961 [8,9] respectively, it has become a well-known etiologic agent of a wide variety of infections, and has assumed increasing importance internationally as a cause of both nosocomial and community-acquired infections. Methicillin-resistant *S. aureus *(MRSA) infections are additional to the burden of methicillin susceptible *S. aureus *(MSSA) and are particularly difficult to treat especially if they are located at anatomical sites, where antibiotic penetration is reduced [10]. Cohort studies of patients with MRSA bacteremia have reported increased morbidity, longer hospital length of stay, and higher costs compared with patients with MSSA bacteremia [4,5,11-15]. In addition, most MRSA are resistant to a number of antimicrobial agents [16].

Although data on the prevalence of staphylococcal infections in Africa are limited, one of the earliest reports of MRSA in the continent was in South Africa [17]. Studies in the 1980s and early 1990s on MRSA in South Africa have also been described [18-20]. However, there is paucity of data on susceptibility patterns of *S. aureus *in South Africa and the prevalence of MRSA in the KwaZulu-Natal (KZN) province is unknown. For practicing physicians, clinical microbiologists and public health officials, knowledge of the local antimicrobial resistance patterns of bacterial pathogens is essential to guide empirical and pathogen specific therapy. The recent reports of *S. aureus *intermediately resistant to vancomycin and teicoplanin in South Africa [21,22] also indicate that information on antibiotic resistance in *S. aureus *is critical for optimal decisions regarding hospital formulary and infection control policies. In addition, characterization of strains is important in understanding the epidemiology of *S. aureus *and evaluating the effectiveness of infection control and antimicrobial prescribing measures [23]. This study reports on the antibiotic susceptibility and characterization of *S. aureus *from clinical samples in KZN, South Africa.

## Methods

### Study areas

The KwaZulu-Natal province is one of the nine provinces in South Africa with a population of about 9.3 million people. A total of 14 provincial hospitals located in seven districts in KZN province, participated in the study. The health institutions included four hospitals in the city of Durban, two, three and five health facilities located in western, southern and northern KZN, respectively. The isolates analyzed in this study were obtained in two phases. The first phase in 2001 (March to August) was part of a survey on the susceptibility pattern of bacterial pathogens obtained from various clinical specimens in health institutions in KZN. The second phase commenced in October 2002 to August 2003. Consecutive non-duplicate *S. aureus *isolates from clinical samples were obtained from the microbiology laboratories of these health institutions.

### Microbiological analysis and identification

A total of 233 isolates were obtained in the two phases. Identification and confirmation of the isolates was conducted by the investigators. They included growth and fermentation on mannitol salt agar, colonial morphology on nutrient agar, Gram stain and positive results for catalase, coagulase and DNase tests. The isolates were preserved in MicroBank (Diagnostic Pro-Lab) and stored at -20°C for further characterization.

### Antibiotic susceptibility testing

The susceptibility testing of isolates to 20 antibiotics was carried out by the disk diffusion method according to the National Committee for Clinical Laboratory Standards (now Clinical Laboratory Standards Institute) guidelines [24]. The antibiotics (Mast Diagnostics) included penicillin (10 U), ampicillin (10 μg), oxacillin (1 μg), gentamicin (10 μg), kanamycin (30 μg), streptomycin (30 μg), neomycin (30 μg), erythromycin (15 μg), clindamycin (2 μg), tetracycline (30 μg), minocycline (30 μg), trimethoprim (2.5 μg), trimethoprim/sulfamethoxazole (25 μg), chloramphenicol (30 μg), ciprofloxacin (5 μg), fusidic acid (10 μg), rifampicin (30 μg), teicoplanin (30 μg), vancomycin (30 μg) and mupirocin (5 μg and 200 μg). Isolates resistant to oxacillin were also screened against methicillin (5 μg) and cefoxitin (30 μg). *S. aureus *ATCC 25923 was the control strain in every test run. Interpretative zone diameters for resistance to fusidic acid, neomycin and streptomycin which are not stated in the CLSI guidelines were considered as follows; ≤ 18 mm – fusidic acid [25], ≤ 16 mm – neomycin, and ≤ 14 mm – streptomycin [16]. The D-test for determining inducible resistance of clindamycin by erythromycin was also performed, in which erythromycin and clindamycin disks were placed 15–18 mm apart. A truncated or blunted clindamycin zone of inhibition (D-shape) indicated inducible resistance. Constitutive resistance was recognized by a clindamycin zone diameter of ≤ 14 mm [26]. Growth to the edge of the 200 μg mupirocin disk and within a 14 mm zone of inhibition with the 5 μg mupirocin disk indicated high and low-level resistance respectively [27]. Furthermore, isolates that expressed phenotypic resistance to oxacillin were screened for intermediate resistance to vancomycin and teicoplanin using the E-test macrodilution method [28].

The resistance rate to each antibiotic was calculated as the number of resistant isolates divided by the total number of isolates. Antibiotyping of MSSA and MRSA was based on the susceptibility patterns to selected antibiotics, representing various classes of antimicrobial agents. They included penicillin (β-lactams), gentamicin (aminoglycosides), erythromycin (macrolides), chloramphenicol (phenicols), tetracycline (tetracyclines), trimethoprim (dihydrofolate pathway inhibitors), rifampicin (ansamycins), ciprofloxacin (fluoroquinolones) and mupirocin. Multiresistance was defined as resistance to penicillin along with at least three classes of antibiotics.

### Molecular detection of the *nuc*, *mecA *and *mupA *genes by PCR

DNA isolation was carried out according to the method previously reported [27]. Isolates resistant to oxacillin, methicillin and cefoxitin by the disk diffusion technique were confirmed as *S. aureus *and MRSA by PCR detection of the *nuc *and the *mecA *genes respectively [29,30]. Low and high-level mupirocin resistant isolates were confirmed by their MIC values (E-test) and the detection of the *mupA *gene [31]. The PCR conditions and detection of PCR products were carried out as described previously [32].

### PCR-RFLP of the coagulase gene

Amplification of the 3' end region of the coagulase gene containing the 81-bp tandem repeats was performed as described previously [33]. *S. aureus *ATCC 25923 served as the positive control in each PCR reaction. Restriction fragment length polymorphisms (RFLPs) of the amplicons were determined by digestion with *Alu*I (Fermentas, UK) by a modification of the protocol previously described [34].

The sizes of the PCR products and of the restriction DNA digests (RFLPs with respect to the overall number of 81-bp tandem repeats) were estimated by comparison with a 100 bp molecular size standard marker, visual inspection and analysis using the GeneTools program (SynGene Bioimaging System). The strains were grouped on the basis of three characteristics of their PCR products, i.e. the presence of one or two PCR products, their size (s), and the *Alu*I restriction digest patterns of the PCR products.

## Results

A total of 233 consecutive non-duplicate *S. aureus *isolates were obtained from clinical samples in 14 health institutions in KZN province, South Africa. Only six isolates (2.6%) were misidentified as *S. aureus*. More than 80% of the total number of isolates was recovered from wound samples, followed by sputum (4%), otitis media (3.1%) and blood samples (2.6%).

The antibiotic susceptibility of *S. aureus *isolates obtained in KZN province, South Africa is described in Table 1. All the isolates were susceptible to teicoplanin, vancomycin and fusidic acid and the proportion of isolates resistant to streptomycin, neomycin, chloramphenicol, ciprofloxacin and mupirocin was less than 10%. Penicillin and ampicillin were the least effective antibacterial agents. In addition, 30% were resistant to erythromycin, clindamycin, trimethoprim and tetracycline, 28.6% to gentamicin, 24.2% to minocycline and 20.3% to rifampicin.

**Table 1:** Antibiotic susceptibility of *S. aureus *isolates (MSSA and MRSA) from KZN province, South Africa

	MSSA n = 166			MRSA n = 61			Total n = 227
Antibiotic	Number of isolates that were:		Resistance rate (%)	Number of isolates that were:		Resistance rate (%)	No/Resistance rate (%)

	S	R		S	R		

Penicillin	19	147	88.6	0	61	100	208 (91.6)
Ampicillin	19	147	88.6	0	61	100	208 (91.6)
Oxacillin	166	0	0	0	61	100	61 (26.9)
Erythromycin	147	19	11.4	11	50	82	69 (30.4)
Clindamycin	148	18	10.8	11	50	82	68 (30.0)
Gentamicin	160	6	3.6	2	59	96.7	65 (28.6)
Streptomycin	166	0	0	42	19	31.1	19 (8.4)
Kanamycin	160	6	3.6	2	59	96.7	65 (28.6)
Neomycin	166	0	0	42	19	31.1	19 (8.4)
Trimethoprim	148	18	10.8	9	52	85.2	70 (30.8)
Trimethoprim/Sulphamethoxazole	148	18	10.8	9	52	85.2	70 (30.8)
Tetracycline	153	13	7.8	6	55	90.2	68 (30.0)
Minocycline	166	0	0	6	55	90.2	55 (24.2)
Teicoplanin	166	0	0	61	0	0	0
Vancomycin	166	0	0	61	0	0	0
Chloramphenicol	165	1	0.6	51	10	16.4	11 (4.8)
Ciprofloxacin	165	1	0.6	50	11	18.0	12 (5.3)
Fusidic acid	166	0	0	61	0	0	0
Rifampicin	165	1	0.6	16	45	73.8	46 (20.3)
Mupirocin (5 μg)	165	1	0.6	46	15	24.6	16 (7.0)
Mupirocin (200 μg)	165	1	0.6	60	1	1.6	2 (0.9)
Methicillin	ND	ND	-	0	61	100	-
Cefoxitin	ND	ND	-	0	61	100	-

KEY

S – sensitive; R – Resistant.

ND: Not Determined

In addition to full susceptibility to teicoplanin, vancomycin and fusidic acid, the MSSA were susceptible to oxacillin, streptomycin, neomycin and minocycline. A total of 18 MSSA were susceptible to all the antibiotics tested. Furthermore, less than 1% of MSSA were resistant to chloramphenicol, ciprofloxacin, rifampicin and mupirocin, while resistance to gentamicin, kanamycin and tetracycline was less than 10%. Of the 19 erythromycin-resistant MSSA, only one isolate exhibited the constitutive macrolide-lincosamide-streptogramin B (MLS_B_) resistance phenotype. The predominant antibiotype among the MSSA was resistance only to penicillin, which was observed in 114 isolates (68.7%) (Table 2). In addition, the proportion of multi-drug resistant MSSA was 3.6% (6 of 166 isolates). Only one MSSA exhibited high-level resistance to mupirocin.

**Table 2:** Antibiotyping of *S. aureus *isolates (MSSA and MRSA) from KZN province, South Africa.

MRSA (n = 61)										No. of MRSA (%)**
Antibiotype*	Resistance patterns									

	PEN	GM	EM	TE	TM	CIP	MUP	CHL	RF	

1	+	+	+	+	+	+	+	+		4 (6.6)
2	+	+	+	+	+	+			+	1 (1.6)
3	+	+	+	+	+		+		+	3 (4.9)
4	+	+	+	+	+			+	+	4 (6.6)
5	+	+	+	+	+	+	+			6 (9.8)
6	+	+		+	+			+	+	2 (3.3)
7	+	+		+	+		+		+	2 (3.3)
8	+	+	+	+	+				+	25 (41.0)
9	+	+	+	+					+	1 (1.6)
10	+	+		+	+				+	5 (8.2)
11	+			+					+	2 (3.3)
12	+	+	+							6 (9.8)

MSSA (n = 166)										

	PEN	GM	EM	TE	TM	CIP	MUP	CHL	RF	

1	+	+	+		+					1 (0.6)
2	+		+	+		+				1 (0.6)
3	+		+	+	+					4 (2.4)
4	+		+		+					1 (0.6)
5	+		+					+		1 (0.6)
6				+	+		+			1 (0.6)
7	+		+	+						2 (1.2)
8	+			+	+					3 (1.8)
9	+	+			+					5 (3.0)
10	+								+	1 (0.6)
11	+			+						2 (1.2)
12	+				+					3 (1.8)
13	+		+							9 (5.4)
14	+									114 (68.7)
15	SUSCEPTIBLE TO ALL ANTIBIOTICS									18 (10.8)

KEY

PEN: Penicillin; GEN: Gentamicin; EM: Erythromycin; TE: Tetracycline; TM: Trimethoprim; CIP: Ciprofloxacin; MUP: Mupirocin (5 μg); CHL: Chloramphenicol; RF: Rifampicin.

* Number of antibiotypes in MRSA and MSSA isolates.

** Percentages in parentheses are based on the total number of isolates in each group (MRSA n = 61; MSSA n = 166).

Resistance to oxacillin, methicillin and cefoxitin was detected in 61 isolates (26.9%). These isolates were confirmed as MRSA by the detection of the *mecA *gene. A total of 48 (78.7%) MRSA isolates were recovered from wound samples, six (9.8%) from sputum, two (3.3%) from otitis media, and one isolate each from blood samples, urine, eye-related infections and endotracheal aspirate. No clinical information was available for one MRSA. Susceptibility testing of MRSA indicated that over 90% of MRSA were resistant to gentamicin and kanamycin, and 31% were resistant to streptomycin and neomycin. Only 9.8% of MRSA were susceptible to tetracycline and minocycline and over 80% were resistant to trimethoprim and trimethoprim/sulfamethoxazole. All the 50 erythromycin-resistant MRSA were positive for inducible MLS_B _resistance using the D-test method. The proportion of MRSA resistant to rifampicin, ciprofloxacin and chloramphenicol was 73.8%, 18% and 16.4%, respectively. Overall, 16 isolates were resistant to mupirocin, of which 14 MRSA exhibited low-level resistance. This resistance phenotype was confirmed by E-test; with MICs values ranging from 8 to 24 μg ml^-1 ^and one MRSA exhibited high-level resistance to mupirocin (>1024 μg ml^-1^).

The antibiotypes of MRSA based on their susceptibility patterns to various classes of antibacterial agents are illustrated in Table 2. MRSA were categorized into 12 antibiotypes, and isolates grouped in type 8 accounted for about 40% of the total number of MRSA. About 87% of MRSA were resistant to at least four classes of antibiotics and more than 40% of MRSA studied were resistant to six classes of antibiotics. In addition, four MRSA were resistant to eight classes of antibiotics. MRSA in antibiotype 1 (resistance to eight classes of antibiotics) were identified in two hospitals in the city of Durban, and one health facility in Pietermaritzburg (Western KZN) and Ngwelezane (Northern KZN). Furthermore, MRSA classified in antibiotype 5 (resistance to seven classes of antibiotics) were noted in two hospitals in the city of Durban and in Northern KZN. MRSA in antibiotype 8 was detected in 12 of the 14 hospitals studied.

**Figure 1:**
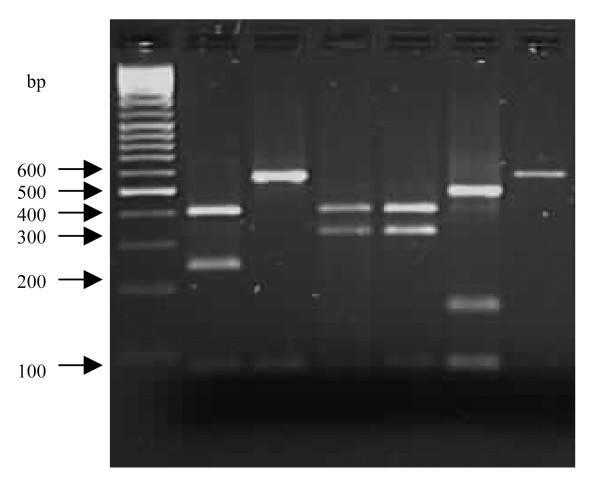
PCR-RFLPs of the coagulase gene in MRSA strains from South Africa showing various types: Lanes 1: 100 bp molecular weight markers. Lane 2: Type 5b; Lane 3: 3a; Lanes 4 and 5: 7b; Lane 6: 7a; Lane 7: 3b

**Figure 2:**
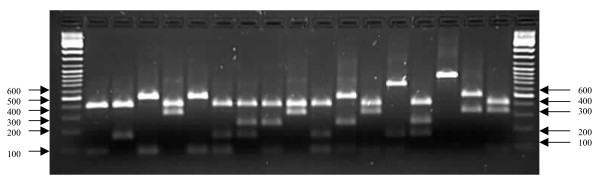
PCR-RFLPs of the coagulase gene in MSSA strains from South Africa showing the various types: Lanes 1 and 19: 100 bp molecular weight markers. Lane 2: Type 1a; Lane 3: 2b; Lane 4: 2a; Lane 5: 3d; Lane 6: 3c; Lane 7: 4a; Lane 8: 4b; Lane 9: 5b; Lane 10: 5d; Lane 11: 5a; Lane 12: 5c; Lane 13: 6a; Lane 14: 7d; Lane 15: 7e; Lane 16: 7g; Lane 17: 7c; Lane 18: 8b

Typing based on PCR-RFLP of the coagulase gene of *S. aureus *isolates from South Africa are illustrated in Figures 1 and 2. PCR products of 98 strains (37 MSSA and 61 MRSA) were analyzed and due to the wide range of sizes of the PCR products, a cut-off value was determined with a limit of ± 20 bp. Among the MRSA strains, a single amplicon of 750 bp was detected in one strain (1.6%), of 850 bp in two strains (3.3%), of 800 bp in 14 (23%), and of 650 bp in 43 strains (70.5%). No PCR product was detected in one MRSA strain. Eleven differently sized PCR products were identified in MSSA strains. PCR amplification of the 3' end of the coagulase gene revealed a single amplicon in 34 of the 37 strains, which ranged between 480 bp and 950 bp. Two PCR amplicons of 400 bp, 750 bp were detected in one strain and of 400 bp, 1000 bp in two strains. A single amplicon of 480 bp was detected in one strain and of 600 bp, 650 bp, 700 bp and 950 bp in two strains respectively. PCR products of 850 bp (five strains), of 750 bp and 900 bp (six strains), and 800 bp (seven strains) were also identified. No PCR product was detected in one MSSA strain. Overall, a single fragment of 650 bp was detected in 45 *S. aureus *strains (MSSA and MRSA), followed by 800 bp in 21 strains, of 750 bp and 850 bp in seven strains and 900 bp in six strains.

A total of 11 distinct RFLP patterns (types 1a–11a) were observed among 96 strains examined after *Alu*I digestion of the PCR products (Table 3). Two additional strains (one MSSA and one MRSA) failed to yield a product with the primers and were therefore classified as twelfth type (12a). The strains belonging to type 7 were subdivided into seven subtypes; group 8 into five subtypes, types 3 and 5 into four subtypes and types 2, 4 and 9 into two subtypes respectively. The 61 MRSA strains were classified into five main RFLP patterns (types 3, 5, 7, 8 and 12) and most of the strains (67.2%) were grouped in subtype 3a. The 37 MSSA strains were categorized into 12 genotypes, and two MSSA in subtype 5b shared similar RFLP patterns with one of the MRSA strains.

**Table 3:** PCR-RFLP of the coagulase gene in MSSA and MRSA strains from South Africa

					*Alu*I restriction fragments									
Type	Molecular weight (± 20 bp)	Total number of strains	*mecA*-positive	*mecA*-negative	81	162	243	324	405	486	567	648	729	810

1a	480	1	0	1	+				+					
2a	600	1	0	1	+					+				
2b		1	0	1		+			+					
3a	650	41	41	0	+						+			
3b		2	2	0							+			
3c		1	0	1	+					+				
3d		1	0	1				+	+					
4a	700	1	0	1	+	+			+					
4b		1	0	1		+	+		+					
5a	750	1	0	1	+	+			+					
5b		3	1	2	+		+		+					
5c		1	0	1	+		+			+				
5d		2	0	2	+			+	+					
6a	750, 400	1	0	1	+			+	+					
7a	800	5	5	0	+	+				+				
7b		7	7	0	+			+	+					
7c		2	2	0				+	+					
7d		2	0	2	+	+						+		
7e		3	0	3		+	+		+					
7f		1	0	1				+		+				
7g		1	0	1										+
8a	850	1	1	0	+	+				+				
8b		1	0	1	+			+	+					
8c		2	0	2		+	+		+					
8d		2	0	2			+			+				
8e		1	1	0				+	+					
9a	900	5	0	5	+	+						+		
9b		1	0	1		+		+		+				
10a	950	2	0	2		+		+		+				
11a	1000, 400	2	0	2		+		+	+	+				
12a	No product	2	1	1										
Total		98	61	37										

The association between antibiotyping and PCR-RFLP of the coagulase gene in MRSA from South Africa is described in Table 4. Nine antibiotypes were noted for MRSA in type 3a (PCR-RFLP: 650 bp; 81, 567 bp) and 88% of strains in the predominant antibiotype 8 were grouped in PCR-RFLP subtype 3a. In addition, five of the six MRSA strains in antibiotype 12 and five of the six MRSA assigned to antibiotype 5 belonged to PCR-RFLP types 7a and 7b respectively. Furthermore, the four strains in antibiotype 1 were equally shared between types 7b and 7c.

**Table 4:** Correlation between PCR-RFLP of the coagulase gene and antibiotyping of MRSA strains from South Africa

Type	(PCR-RFLP coagulase gene) ± 20 bp (number of strains)	Antibiotype (number of strains)								
		PEN	GM	EM	TE	TM	CIP	MUP	CHL	RF
3a	650 (81, 567) (41)	+	+	+	+	+	+			+ (1)
		+	+	+	+	+		+		+ (3)
		+	+	+	+	+			+	+ (4)
		+	+		+	+			+	+ (2)
		+	+		+	+		+		+ (2)
		+	+	+	+	+				+ (22)
		+	+	+	+					+ (1)
		+	+		+	+				+ (4)
		+			+					+ (2)
3b	650 (567) (2)	+	+	+	+	+				+ (1)
		+	+		+	+				+ (1)
5b	750 (81, 243, 405) (1)	+	+	+	+	+				+ (1)
7a	800 (81, 162, 486) (5)	+	+	+						(5)
7b	800 (81, 324, 405) (7)	+	+	+	+	+	+	+	+	(2)
		+	+	+	+	+	+	+		(5)
7c	800 (324, 405) (2)	+	+	+	+	+	+	+	+	(2)
8a	850 (81, 162, 486) (1)	+	+	+						(1)
8e	850 (324, 405) (1)	+	+	+	+	+	+	+		(1)
12a	No product (1)	+	+	+	+	+				+ (1)

## Discussion

Antimicrobial resistance has been noticed as one of the paramount microbial threats of the twenty-first century [35]. *S. aureus *has always been a stumbling block for anti-microbial chemotherapy and the introduction of new classes of antimicrobial agents is usually followed by the emergence of resistant forms of this pathogen [16,36]. Therefore, surveillance on the antimicrobial susceptibility patterns of *S. aureus *is of utmost importance in understanding new and emerging resistance trends and in the management of both hospital and community-acquired infections.

In this study, all the isolates were susceptible to teicoplanin, vancomycin and fusidic acid, while penicillin and ampicillin were the least effective antimicrobial agents. Although the disk diffusion technique utilized in this study for the determination of susceptibility to vancomycin and teicoplanin is unreliable due to its low sensitivity [37], isolates that exhibited resistance to oxacillin were screened for intermediate resistance to vancomycin and teicoplanin using the E-test macrodilution method [28]. None of the MRSA demonstrated resistance to these antibiotics. The susceptibility patterns of *S. aureus *isolates in this study were compared with data from an international multi-centre study, in which 21 laboratories in 18 countries (including South Africa) participated [38]. The full susceptibility of *S. aureus *to fusidic acid observed in this study agreed with data from the multi-centre survey, indicating that fusidic acid is an excellent and effective agent for the treatment of *S. aureus *infections in South Africa. However, monotherapy with fusidic acid has been associated with the emergence of resistance; therefore it is usually combined with another antistaphylococal agent (beta-lactams, rifampicin or glycopeptides) to minimize the emergence of fusidic-acid resistant strains [39]. The prevalence of *S. aureus *resistance to rifampicin, erythromycin, clindamycin, and tetracycline noted in this study was also comparable with data from the multi-centre study (South Africa). However, resistance to ciprofloxacin was lower in this investigation (5.3% vs 29%) compared with the multi-centre survey.

Since the emergence of the first clinical isolate of MRSA was reported in 1961, this pathogen has posed challenges in the treatment of infections in which its characteristic nature of multi-drug resistance restricts the options to treat infections [40-42]. One of the earliest reports of MRSA in South Africa was reported in a Durban hospital [17]. Of the 227 *S. aureus *isolates obtained in this study in the KZN province of South Africa, 61 (26.9%) were confirmed as MRSA. The prevalence of MRSA was lower than previous reports in major cities in South Africa such as Johannesburg and Cape Town, which ranged between 33% and 43% [7,38,43-45]. However, the prevalence of MRSA in hospitals within the city of Durban was 34%, which is comparable with previous data in major cities in South Africa. A limitation in this study was that a comparative analysis of MRSA based on the geographic location of the health institutions could not be determined due to the low and varying numbers obtained in the various health institutions. Based on the level of resistance and grouping as described in the multi-centre study [38], *S. aureus *resistance to oxacillin along with gentamicin, kanamycin, tetracycline, minocycline, erythromycin, clindamycin, rifampicin, and trimethoprim could be considered to be of concern in KZN province, South Africa.

There was a relationship between methicillin resistance and resistance to other antibiotics as noted in previous investigations [16,38,43,46]. This study also supports the observation of a relationship between oxacillin and aminoglycoside resistance in *S. aureus *[16,47]. More than 90% of MRSA were resistant to gentamicin and kanamycin whereas less than 4% of MSSA were resistant to these aminoglycosides. While the frequency of MRSA resistance to the tetracyclines (tetracycline and minocycline) was high (90.2%), in-vitro resistance to minocycline was not observed in MSSA and only 7.8% were resistant to tetracycline. Resistance to rifampicin by MSSA was less than 1% while MRSA resistance was 74%.

A major problem in the treatment of *S. aureus *infections is the ability of this pathogen to be resistant to a number of antibiotics. In the last few years, understanding of the genetic basis for methicillin resistance has advanced significantly. Staphylococcal cassette chromosome *mec *(SCC *mec*) elements are, so far, the only vectors described for the *mecA *gene encoding resistance in staphylococci [48]. As well as resistance to all beta-lactams, the SCC *mec *can encode resistance to bleomycin, macrolide-lincosamide-streptogramin B, aminoglycosides (tobramycin, amikacin), and spectinomycin [49]. Multi-resistant MRSA has been reported to be relatively high in African countries including Morocco, Kenya, Nigeria and Cameroun [50], but their antibiotypes were not determined. In this study, MRSA were grouped into 12 antibiotypes and 87% were resistant to at least four classes of antibiotics. In addition, isolates belonging to antibiotype 8 were identified in 12 of the 14 hospitals, indicating that MRSA with this resistance phenotype appears to be widespread in KZN. MRSA classified in antibiotypes 1 and 5 (resistance to eight and seven classes of antibiotics respectively) were identified in health institutions located in Durban, Pietermaritzburg (Western KZN) and Ngwelezane (Northern KZN). This study confirms the multi-resistant nature of healthcare-associated MRSA as reported by previous investigations from various regions of the world [16,46,51,52]. It also indicates that treatment of infections caused by healthcare-associated MRSA may be difficult in this province, as there are reduced antimicrobial options, which could lead to substantial rates in morbidity and mortality in hospital patients and increased health cost.

About 65% of *S. aureus *susceptible to erythromycin and clindamycin were MSSA, while the inducible MLS_B _phenotype was detected in 10.8% of MSSA and 82% of MRSA respectively. A recent survey in Pennsylvania, USA, observed that 68% of MSSA and 12.3% of MRSA were D-test positive [53]. In our study, the constitutive MLS_B _phenotype was identified in one MSSA isolate, but absent in all the MRSA. This trend is in contrast to the report in Korea whereby 24% of MSSA and 86% of MRSA exhibited constitutive resistance [16]. Furthermore, the constitutive MLS_B _phenotype is known to be a common feature among MRSA isolates in Belgium [54] and Greece [55]. These observations indicate that the incidence of constitutive and inducible MLS_B _resistance in staphylococcal isolates varies by geographic region. The D-test was demonstrated, like previous studies, to be a simple and reliable method to detect inducible resistance to clindamycin. In January 2004, the National Committee for Clinical Laboratory Standards (now CLSI) published a procedure for clindamycin induction testing [56]. The clinical microbiology laboratories in South Africa should consider routine testing and reporting of inducible clindamycin resistance in *S. aureus*. This is to ensure that clinicians can rely on clindamycin test results and be informed about the possibility of clindamycin treatment failure in patients with infections caused by inducibly resistant isolates. The proportion of MRSA with the inducible MLS_B _phenotype (82%) indicates that clindamycin may not be a theraupeutic option for the treatment of an infection attributed to an inducibly resistant MRSA. If clindamycin is used for treatment of infections with MLS_B_i-positive isolates, close follow-up and monitoring of failure or relapse is needed. However, in more severe infections, the presence of the MLS_B_i phenotype should preclude the use of clindamycin.

Our study observed a high prevalence of rifampicin resistance (73.8%) among the MRSA, suggesting that this trend may be increasing worldwide. Rifampicin resistance in *S. aureus *has been reported in Australia [57], United Kingdom [58], Malaysia [59], Turkey [60] and Poland [61]. Furthermore, a recent study on MRSA in eight African countries noted that the prevalence of rifampicin resistance was high with the exception of two countries (Morocco and Kenya) [50]. Resistance to rifampicin has been reported to be a common trend among *S. aureus *in South Africa [44] and clinical isolates of *Mycobacterium tuberculosis *[62]. This could be attributed to the widespread use of this antimicrobial agent. Interestingly, a high level of rifampicin resistance has also been observed in environmental isolates of members of the family *Enterobacteriaceae *in Northern KZN [63]. These facts indicate the severity of rifamipicin resistance in both clinical and environmental bacteria in this province and probably in South Africa as a whole.

Parenteral glycopeptides (vancomycin and teicoplanin) are the mainstay of therapy for MRSA infections. However, rifampicin, fusidic acid, ciprofloxacin and trimethoprim-sulfamethoxazole (TMP-SMX) are widely used oral agents that have demonstrated consistent in-vitro activity and are recommended in the therapy of MRSA infections [16,57,64]. Combination therapy with two oral agents is thought to be important to decrease the risk of selecting for mutants during therapy of MRSA infections [64-67]. In Australasia, rifampicin and fusidic acid are the usual combination to treat MRSA infections [64,68], while in the United States, TMP-SMX is claimed to be widely used, with or without rifampicin, for MRSA infections that are not life-threatening [69]. This study observed that 73.8% and 85.2% of MRSA were resistant to rifampicin and TMP-SMX respectively. Although we did not investigate the level of usage regarding TMP-SMX and rifampicin in these hospitals, combination treatment with these antibacterial agents would be unreliable in KZN, South Africa.

Resistance to mupirocin was detected in 16 *S. aureus *isolates and 94% were MRSA. The prevalence of mupirocin resistance in this study (7%) was higher than a previous study (2%) in South Africa [38]. However, high-level mupirocin resistance by *S. aureus *in our study was lower than surveys conducted in Greece [70,71], South Korea [72] and Poland [73]. One MSSA and one MRSA were *mupA *positive, while all the isolates with low-level mupirocin resistance (*mupA *negative) were MRSA. This trend suggests that mupirocin resistance in *S. aureus *is an emerging feature in this province, particularly in Durban. To the best of our knowledge, this is the first report of *S. aureus *resistance to mupirocin in this province. Inspite of the small amount of data on mupirocin resistance in MSSA, it is suggested that MRSA along with MSSA should be routinely tested in clinical microbiology laboratories in this province so that resistant isolates could be detected early, and to facilitate the prompt institution of infection control measures.

Coagulase is produced by strains of *S. aureus *[74]. Its production is the principal criterion used in the clinical microbiology laboratory for the identification of *S. aureus *in human infections [75]. The coagulase gene has been a target for PCR genotyping in which size and DNA restriction endonuclease site polymorphism within the *coa *gene have been utilized for PCR-RFLP analysis [76]. Coagulase gene typing has been reported to be an attractive method for clinical laboratories because of its ease and speed, and has been widely used in genotyping of clinical *S. aureus *isolates [33,77-83].

Results of coagulase gene typing demonstrated that the MRSA and MSSA strains from South Africa were classified into four and eleven RFLP patterns, respectively. However, two strains (one MSSA and one MRSA) failed to yield a PCR product with the primers. The inability for a gene product to be obtained could be due to sequence variations at the sites targeted by the primers, as described by some investigators [75,83,84]. A total of 67% of MRSA were classified into the subtype 3a, indicating that strains with this profile were predominant in health institutions in KZN province, South Africa. In contrast, the MSSA strains were diverse and none of the RFLP patterns could be considered as a predominant group. This finding is similar to previous studies [33,78]. It also indicates that genomic variation was lower in MRSA than in MSSA strains. The ability of the PCR-RFLP of the coagulase gene to differentiate between MRSA and MSSA from South Africa was also observed. The MSSA and MRSA strains did not share similar PCR-RFLP patterns in types 3, 7 and 8, while none of the MRSA strains were identified in types 1, 2, 4, 6, 9, 10 and 11. The RFLP patterns of the MRSA strains were unique and distinct from the MSSA strains, but two MSSA in subtype 5b shared similar PCR-RFLP patterns with one of the MRSA strains. The ability of the PCR-RFLP to distinguish MSSA from MRSA strains has been reported [79], and offers an attractive option to be considered in the epidemiological analysis of *S. aureus *in South Africa. Some degree of correlation between the two typing methods was observed. A good correlation between antibiotyping and PCR-RFLP of the coagulase gene was observed in identifying strains within antibiotype 12. Only one strain in antibiotype 12 (PCR-RFLP group 8a) produced a differently sized amplicon by PCR detection of the coagulase gene, but the RFLP pattern was similar with the rest of the strains in PCR-RFLP group 7a. All the ten multi-drug resistant MRSA strains assigned in antibiotype groups 1 and 5 produced similar PCR-RFLP patterns indicating that they were closely related.

## Conclusion

This study has provided baseline information in assisting physicians, clinical microbiologists and public health officials on critical issues regarding empirical and pathogen specific therapy. The isolation of multi-resistant MRSA in various health facilities, the wide dissemination of MRSA grouped in antibiotype 8 and the emerging trend of mupirocin resistance indicate that adequate steps in limiting spread are urgently needed. Continuous surveillance on resistance patterns of *S. aureus *in understanding new and emerging trends is of utmost importance. PCR-RFLP of the coagulase gene represents an attractive tool for the rapid initial genotyping of *S. aureus *in South Africa.

## Competing interests

The author(s) declare that they have no competing interests.

## Authors' contributions

AOS participated in the collection, susceptibility pattern and characterization of the *S. aureus *isolates. JL supervised the project. All the authors participated in the preparation of the final manuscript.

## Pre-publication history

The pre-publication history for this paper can be accessed here:


